# Phytochemicals: A Multitargeted Approach to Gynecologic Cancer Therapy

**DOI:** 10.1155/2014/890141

**Published:** 2014-07-01

**Authors:** Lee Farrand, Se-Woong Oh, Yong Sang Song, Benjamin K. Tsang

**Affiliations:** ^1^Yuhan Research Institute, Yuhan Corporation, Giheung-gu, Yongin-si 416-1, Republic of Korea; ^2^WCU Biomodulation, Department of Agricultural Biotechnology, Seoul National University, Seoul 151-921, Republic of Korea; ^3^Cancer Research Institute, Seoul National University College of Medicine, Seoul 110-799, Republic of Korea; ^4^Department of Obstetrics and Gynecology, Seoul National University College of Medicine, Seoul 110-744, Republic of Korea; ^5^Department of Obstetrics & Gynaecology, University of Ottawa, Ottawa, ON, Canada K1H 8L6; ^6^Department of Cellular & Molecular Medicine, University of Ottawa, Ottawa, ON, Canada K1H 8L6; ^7^Interdisciplinary School of Health Sciences, University of Ottawa, Ottawa, ON, Canada K1H 8L6; ^8^Chronic Disease Program, Ottawa Hospital Research Institute, General Campus, Critical Care Wing, 3rd Floor, Room W3107, 501 Smyth Road, Ottawa, ON, Canada K1H 8L6

## Abstract

Gynecologic cancers constitute the fourth most common cancer type in women. Treatment outcomes are dictated by a multitude of factors, including stage at diagnosis, tissue type, and overall health of the patient. Current therapeutic options include surgery, radiotherapy, and chemotherapy, although significant unmet medical needs remain in regard to side effects and long-term survival. The efficacy of chemotherapy is influenced by cellular events such as the overexpression of oncogenes and downregulation of tumor suppressors, which together determine apoptotic responses. Phytochemicals are a broad class of natural compounds derived from plants, a number of which exhibit useful bioactive effects toward these pathways. High-throughput screening methods, rational modification, and developments in regulatory policies will accelerate the development of novel therapeutics based on these compounds, which will likely improve overall survival and quality of life for patients.

## 1. Introduction

Gynecologic cancers are malignant neoplasms of the female reproductive system, the most common of which are endometrial, ovarian, and cervical cancers. Together, they constitute the fourth most common cancer type in women, with approximately 82,000 diagnosed in the USA annually [1]. Treatment outcomes for endometrial and cervical cancers are relatively more effective, due to the availability of more definitive screening methods and a faster onset of symptoms that generally prompt earlier intervention. In contrast, ovarian cancer is the most deadly, with more women dying of the disease than all other types of gynecologic cancer combined [2]. This can be attributable to a lack of symptoms and detectable biomarkers, frequently resulting in late-stage diagnoses.

## 2. Therapy and Chemoresistance

First-line treatment strategies for gynecologic cancers are administered depending on the stage and malignant cell type involved, but surgical intervention and chemotherapeutic agents such as paclitaxel and cisplatin-based derivatives are frequently included. Endometrial cancers are most effectively treated with surgery, via hysterectomy [3]. Clinical studies have demonstrated the efficacy of less aggressive surgical approaches, when decisions take into account lower grades outlined by the International Federation of Gynecology and Obstetrics [4]. In contrast, more advanced endometrial cancers can only be optimally debulked in 44–72% of cases [5, 6]. Neoadjuvant chemotherapy in such cases has yielded some positive outcomes [7], but the only large randomized trial involving chemotherapy for endometrial cancer found no difference in survival between the groups that received doxorubicin or no further therapy following regular surgical intervention [8]. Similarly, the efficacy of radiotherapy remains controversial, with ambiguous and conflicting lines of evidence [9, 10].

Most cervical cancers are squamous cell carcinomas arising from the epithelial cells lining the cervix. Treatment strategies include radical surgery or radiotherapy; however, extensive clinical studies have shown that no treatment of choice exists for early stages of the disease [11]. A combination of surgery and radiotherapy results in higher morbidity, and the optimal therapy for each individual patient is reliant on clinical factors such as age and histological type. For advanced stages of the disease, pelvic radiation has become the currently accepted gold standard [12]. In addition, a combination of histone deacetylase inhibitor (vorinostat) and proteasome inhibitor (bortezomib) has been shown to significantly retard cervical tumor growth in a xenograft model, although such an approach in a clinical setting has yet to be attempted [13].

In contrast, cisplatin (CDDP:* cis*-diamminedichloroplatinum) and its derivatives are considered first-line treatments for ovarian cancer, following surgical debulking [14]. In most cases, however, recurrent disease emerges that fails to respond to further chemotherapy. This phenomenon is referred to as chemoresistance and often signals the end of the road in terms of viable treatment options. Chemoresistance arises from the dysregulation of signaling factors responsible for inducing cell death [15]. Current standards of treatment using chemotherapy primarily focus on ovarian cancer; however, all of the gynecologic cancer types may be susceptible to novel chemotherapeutic approaches. One concern that remains clear is that the current therapeutic options of radiotherapy, surgery, and chemotherapy for gynecologic cancers are insufficient for current patient needs. The severity of side effects and frequent development of infertility posttherapy necessitate the development of more sensitive and personalized strategies for higher standards of treatment.

## 3. Phytochemicals

Phytochemicals are a broad class of molecules with bioactive properties that are derived from botanical sources. In recent years, a growing number of studies have uncovered a plethora of potential applications for phytochemicals in signaling pathways related to cancer [16]. Bioactive compounds that can inhibit or antagonize factors that are dysregulated in malignant cells have the potential to enhance the effects of conventional therapy or be developed into a stand-alone therapeutic in their own right. One major advantage for the use of phytochemicals over synthetic compounds, in many cases, is their historical presence in the human diet. Due to this evolutionary exposure, severe adverse events are conceivably less likely to arise in therapeutic settings when compared to synthetic compounds that are entering the human body for the first time. Modern high-throughput screening techniques can also facilitate the screening of fractionated separations of plant extracts containing thousands of phytochemicals, while synthetic libraries require each candidate to be engineered separately. Some phytochemicals also exert influences on multiple targets within a common oncogenic signaling pathway [17]. Many oncogenic signaling pathways are shared by malignant cells across different tissue types, due to common functional requirements for sustained survival and proliferation. Therefore, phytochemicals that exhibit anticancer activity in one cell type may have potential for application in treating a wider range of cancers.

## 4. Molecular Mechanisms of Phytochemical Action in Cancer Prevention

The science of cancer prevention receives relatively little attention when compared to the field of cancer therapy. Whether a result of market forces or a lack of experimental precedent in developing preventative approaches is unclear. However, environmental factors including tobacco smoking and a sedentary lifestyle are known to contribute to a higher risk of many cancers. Epidemiological evidence also suggests that dietary behavior significantly influences the prevalence of specific cancer types in any given population [18]. A diet high in fruits and vegetables appears to broadly reduce cancer risk, and this can be at least partially attributable to the bioactivity of phytochemicals [19]. Perhaps the most recognized example is resveratrol, a phytoalexin found in the skins of red grapes. Resveratrol exhibits a number of striking bioactive properties beneficial for human health, including antitumor activity [20].

Luteolin is a flavonoid present in cruciferous vegetables that exhibits cancer chemopreventive activity. It inhibits protein kinase C*ɛ* and Src kinase activities, both of which have been implicated in oncogenic signaling [21]. Other phytochemicals may exert chemopreventive activities by targeting alternative hallmarks of cancer such as angiogenesis and inflammation. Myricetin is one of the major phytochemicals present in onions and berries and has been found to inhibit angiogenesis via the inhibition of PI3K and the suppression of matrix metalloproteinases responsible for vascular growth [22]. These findings have been supported by a mouse model of angiogenesis, in which myricetin topical treatment was sufficient to suppress UV-B induced blood vessel formation. Meanwhile, apigenin (another abundant flavonoid found in onions and berries) has been shown to counteract inflammatory processes via direct binding to cyclooxygenase 2, thereby suppressing downstream events [23]. Apigenin, as well as chalcone (a pigment of petunia flowers), can regulate MAPK pathways in endometrial cancer cells via selective action on activator protein-1 [24]. Similarly, sulforaphane (found in cruciferous vegetables) has been demonstrated to trigger cell cycle arrest in cervical cancer cells when treated at low concentrations [25].

## 5. Phytochemical-Based Approaches to Overcoming Chemoresistance

Chemoresistance arises in cancer cells via the downregulation of tumor suppressors and the stabilization or activation of cell survival factors [26]. Mutation, overexpression, or gene deletions are responsible for dysregulating apoptosis signal pathways. The identification and targeting of such dysregulation with bioactive compounds may therefore represent a viable strategy for improving chemosensitivity.

**Figure 1:**
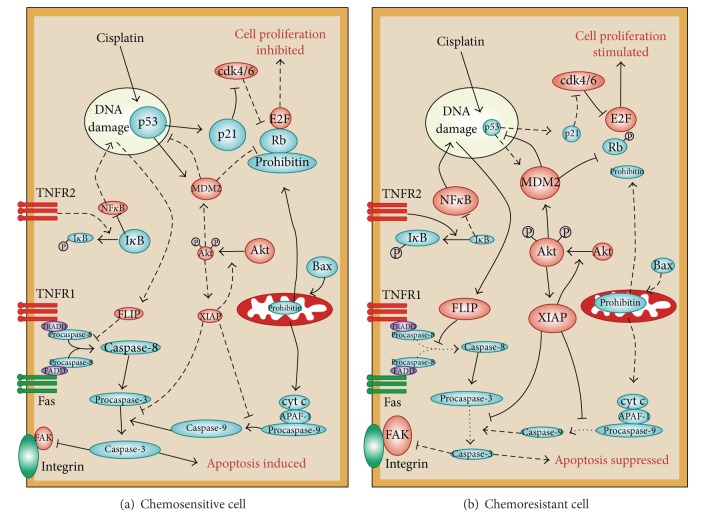
A hypothetical model of chemoresistance in human ovarian cancer cells. In a chemosensitive ovarian cancer cell (a), cisplatin activates p53, leading to upregulation of proteins promoting cell cycle arrest, such as p21, and of proapoptotic proteins such as Bax and Fas. This activates both the intrinsic (mitochondrial) and the extrinsic (death-receptor) apoptotic pathways, the overall result of which is the activation of the execution caspase-3 (and caspase-7, not shown). In these cells, cell survival mediators such as Xiap, Akt, and Flip (shown in red) are downregulated or are in their inactive state. In chemoresistant cells (b), increased p53 ubiquitination by MDM2 results in the maintenance of low levels of p53, despite the presence of cisplatin. Moreover, cisplatin fails to downregulate Xiap, thereby maintaining an active state of the PI3K/Akt pathway. In addition, binding of TNFR2 by TNF*α* leads to upregulation of FLIP through the NF-*κ*B pathway, thus inhibiting the proapoptotic actions of the cytokine through TNFR1. Overall, as a consequence of a failure to activate the caspase cascade in response to the chemotherapeutic agent, these cells have lost their capacity to undergo apoptosis and thus became chemoresistant. Taken from [15].

The PI3K/Akt pathway is frequently overexpressed or activated in a number of cancers (Figure 1). The downregulation of Akt sensitizes chemoresistant ovarian cancer (OVCA) to CDDP-induced apoptosis, at least in part, by modulating cisplatin-induced, p53-dependent ubiquitination of Fas-associated death domain-like interleukin-1 beta-converting enzyme- (FLICE-) like inhibitory protein (FLIP) [27, 28]. Akt inhibition has been shown to sensitize chemoresistant ovarian cancer cells to paclitaxel [29], while other studies have shown that its downregulation stabilizes the p53-inducible protein phosphatase PPM1D, increasing its content in response to cisplatin challenge. In chemoresistant cells with high Akt expression, PPM1D stability is enhanced in response to protein synthesis inhibition, which is significantly decreased in the chemosensitive response [30]. The caspase-independent apoptosis pathway, regulated by the activity of apoptosis-inducing factor (AIF), is also influenced by Akt action through its attenuation of AIF nuclear translocation [31]. AIF is negatively regulated by the X-linked inhibitor of apoptosis protein (XIAP), another determinant of chemoresistance that is also stabilized by Akt [32, 33]. XIAP, in turn, can regulate Akt activity and caspase-3-dependent cleavage during CDDP-induced apoptosis [34]. The tumor suppressor p53 also plays a central role in apoptosis and its phosphorylation at serine residues 15 and 20 stabilizes it by preventing association with murine double minute 2 (MDM2) [28, 35]. Many lines of evidence show that a functional p53 significantly affects the capacity of cancer cells to undergo apoptosis [27, 36, 37]. It has been demonstrated that p53 promotes the ubiquitination and subsequent proteasomal degradation of FLIP by promoting its interaction with the E3 ligase Itch [38].

**Figure 2:**
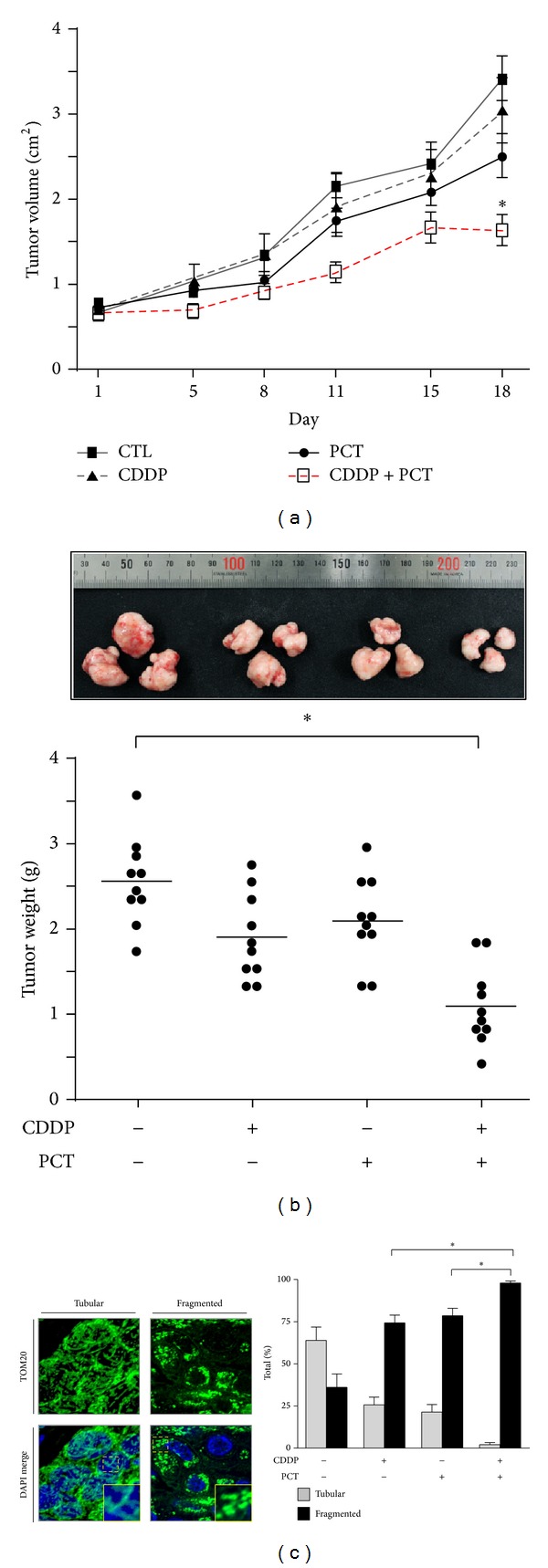
Effects of piceatannol and CDDP treatment on tumor growth in a mouse model of OVCA. (a) Effect of CDDP (1.8 mg/kg, once per week) and piceatannol (20 mg/kg, 5 times per week) on tumor volume. Tumors were formed by subcutaneous insertion of 1 × 10^6^ OV2008 cells embedded in matrigel into the hind flanks of athymic nude mice. Tumors were measured over 18 days for the intervals indicated and volume was calculated using the equation *V* = *π*/6(*l* × *h* × *w*). (b) Measurements of tumor weight on the day of sacrifice (*P < 0.05). (c) Effect of CDDP and piceatannol treatment on mitochondrial morphology in recovered tumors. Taken from Farrand et al. [42].

A crucial step in the induction of apoptosis is mitochondrial outer membrane permeabilization (MOMP). Proapoptotic Bcl-2 family members (including Bax and Bak), as well as the BH3-only proteins (Bid, Bim, and PUMA) permeabilize the membrane after activation [39]. When challenged with cisplatin, p53 has been shown to translocate to the mitochondria and facilitate MOMP by interacting with membrane proteins that mediate pore formation [15, 37]. This results in the release of cytochrome c and second mitochondria-derived activator of caspases, leading to cell death. Dynamin-related protein 1 (Drp1) is a cytosolic GTPase responsible for the process of mitochondrial fission and is activated by cytosolic changes in calcium levels via its regulator, calcineurin [40]. Fission precedes cellular apoptosis in the majority of cases, and the speed at which it occurs can directly influence its induction [41]. Studies have shown that the phytochemicals piceatannol and piperlongumine (found in red grapes and the long pepper, resp.) can enhance cisplatin-induced apoptosis through enhanced levels of Drp1-dependent mitochondrial fission [42, 43]. Piceatannol, a natural stilbene and a metabolite of resveratrol, enhances cisplatin sensitivity in ovarian cancer, by increasing the p53-mediated expression of the proapoptotic protein NOXA, XIAP degradation via the ubiquitin-proteasome pathway, and promoting caspase-3 activation. These effects are also associated with increases in Drp1-dependent mitochondrial fission, a step that appears to improve the induction of apoptosis. A xenograft mouse model has shown that these events translate into additive reductions in tumor size when treatment includes both cisplatin and piceatannol in combination (Figure 2).

Curcumin has been shown to sensitize cervical cancer cells to paclitaxel treatment* in vivo *[44]. Curcumin exerts its effect via downregulation of the NF-*κ*B, MAPK, and Akt pathways and, in combination with paclitaxel, induces a synergistic reduction in tumor incidence as well as tumor volume in a xenograft model using NOD-SCID mice. Moreover, preexposure of cervical cancer cells to curcumin was found to potentiate paclitaxel sensitivity in 3-methylcholanthrene-induced cervical carcinoma models. Similarly, both apigenin and emodin (a purgative resin found in Himalayan rhubarb) have been shown to control Fas and TRAIL sensitivity in endometrial cancer cells via the inhibition of casein kinase [45].

**Figure 3:**
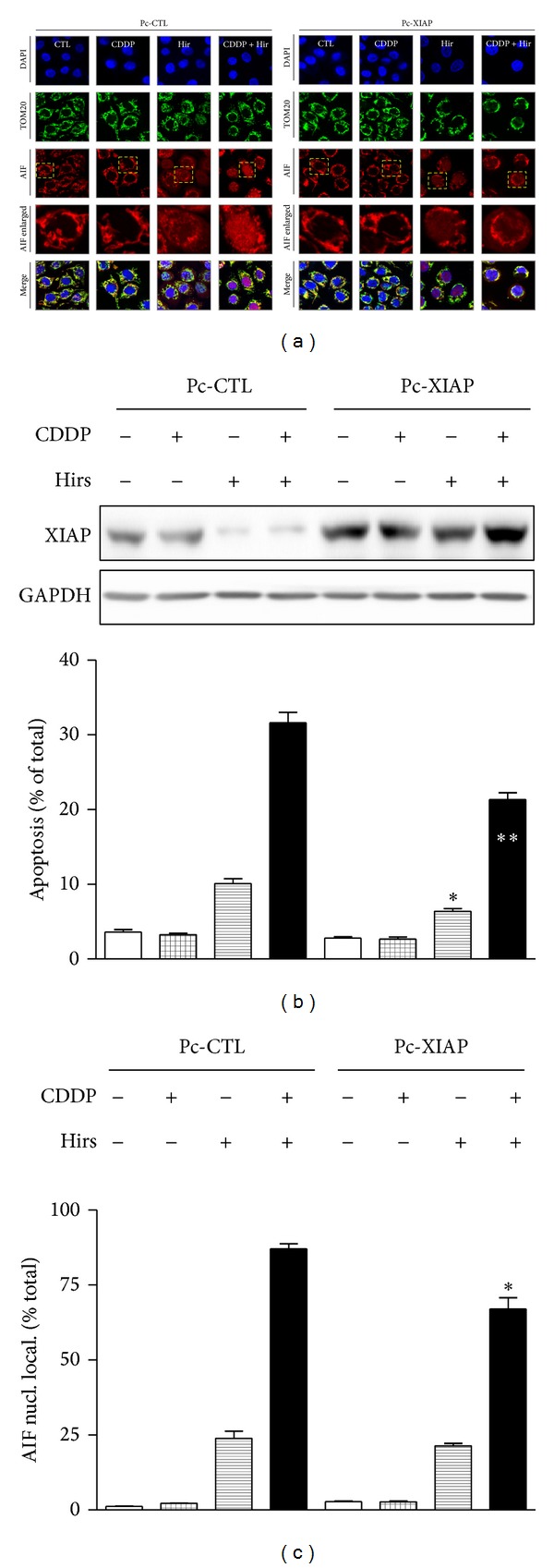
Hirsutenone-facilitated CDDP-induced apoptosis in chemoresistant OVCA cells is mediated by AIF and by suppressed XIAP-AIF interaction. (a) Effects of hirsutenone (10 *μ*M) and CDDP (10 *μ*M) treatment (12 h) on AIF nuclear translocation, in the presence and absence of XIAP overexpression. Chemoresistant cells were transfected with XIAP overexpression constructs (Pc-XIAP, 0.1 *μ*g, 48 h) or GFP control constructs (Pc-GFP, 0.1 *μ*g, 48 h), prior to treatment. Blue: DAPI, red: AIF, and green: TOM20 (mitochondrial membrane marker). (b) Effects of XIAP overexpression on apoptosis induced by hirsutenone (10 *μ*M) and CDDP treatment (10 *μ*M, 24 h). Chemoresistant cells were transfected with XIAP overexpression constructs (Pc-XIAP, 1 *μ*g, 48 h) or GFP control constructs (Pc-GFP, 1 *μ*g, 48 h), prior to treatment. (c) Quantification of AIF nuclear localization data shown in (a). Nuclear signal was quantified using Image J software (*P < 0.05; **P < 0.01 versus respective DMSO control (CTL)). Taken from [46].

Of the gynecologic cancers, ovarian and cervical cancers have received the most attention in terms of phytochemical approaches to overcome chemoresistance. Hirsutenone, a diarylheptanoid from the bark of* Alnus hirsuta,* has been shown to sensitize chemoresistant ovarian and cervical cancer cells to cisplatin [46]. Hirsutenone activates p53 via phosphorylation at Ser 15 in cells with wild type-p53 and also has significant effects in p53-null and p53-mutant cell lines. CDDP-dependent apoptosis in chemoresistant cells was associated with ubiquitin/proteasome-mediated degradation of XIAP and enhancement of AIF translocation from the mitochondria to the nucleus. These effects appeared to in part be regulated by Akt, linking hirsutenone-dependent PI3K inhibition with its downstream apoptotic effectors AIF and XIAP (Figure 3). Other phytochemicals with an ability to overcome chemoresistance in ovarian cancer include the citrus flavonoid tangeritin [47], the turmeric compound curcumin [48], and the resveratrol [49].

To overcome the problem of chemoresistance, researchers have begun to focus on the identification of novel targets for inhibition by small molecules. Smac mimetics are synthetic compounds that mimic the role of second mitochondrial activator of caspases protein by binding to and inhibiting the activity of IAP family members like XIAP [50]. Studies have shown that such compounds can induce apoptosis in chemoresistant ovarian cancer cells by potentiating ligand-mediated death pathways [51]. New promising targets that have yet to be validated in clinical settings include the ubiquitin specific protease 8 (USP8) and pyruvate kinase M2 (PKM2) [52]. USP8 regulates the expression of receptor tyrosine kinases responsible for downstream activation of oncogenic signaling pathways including the PI3K/Akt and MAPK s pathways, and its inhibition with a small molecule has been shown to selectively kill cancer cells. PKM2 regulates aerobic glycolysis in tumor cells, providing the metabolic advantage required for rapid proliferation. Its knockdown with short hairpin RNA leads to a reversal of the Warburg effect and inhibits tumor growth in a xenograft model [53].

High-throughput screening of phytochemical libraries may identify potent compounds to inhibit such novel targets and overcome chemoresistance.

Recently there have also been reports that the US FDA is reconsidering its regulatory framework for the approval of novel cancer therapeutics [54]. Instead of the conventional approach of approving cancer drugs for a specific indication, there may be progress toward approval based solely on the molecular pathway that a drug is targeting. If this framework is implemented, considerable flexibility will be conferred to the pharmaceutical industry in the development and clinical testing of new drug candidates. The heterogeneity present in tumor cell populations also justifies more versatility in the choice of therapeutic regimen available to physicians.

## 6. Phytochemical Analogues and Chemical Modifications for Greater Efficacy

Some phytochemicals have multiple molecular targets, and such properties are not limited to application in gynecologic cancers alone. Geraniol is an effective plant-based mosquito repellant present in a number of essential oils including citronella. This acyclic monoterpene has been shown to independently induce apoptosis and autophagy via the inhibition of Akt and the activation of AMPK. It has also been demonstrated that the combined effect of Akt inhibition and AMPK signaling is more potent at suppressing prostate cancer cell growth than either action alone [55]. Moreover, when treated in combination with docetaxel, geraniol markedly improved chemosensitivity in a xenograft model [56].

A close structural analogue of geraniol is the more widely known compound menthol. Of particular note, menthol has been widely used in foods, cosmetic products, and topical therapeutic creams for centuries. Studies have shown that menthol binds and activates the TRPM8 Ca(2+)-permeable channel that exhibits abnormal expression patterns in a number of cancer types [57]. Menthol has also been found to markedly downregulate activity of the polo-like kinase 1 (PLK1), thereby inhibiting progression of the G2/M phase in malignant cells [58].

In some cases, phytochemicals can be used to demonstrate proof of concept and to contribute to the development of rationally designed therapeutics. Although not structurally related to menthol, icilin is a synthetic superagonist that was rationally designed to target the same TRPM8 channel as menthol and also produces an extreme sensation of cold. Interestingly, icilin induces G1 arrest in the absence of cell death, via activation of JNK and p38 kinase pathways [59]. Imidazole is another organic compound present in many important biological molecules. SK&F 96365 is a synthetic imidazole derivative that also targets TRP calcium channels. Unexpectedly, its off-target effects promote necrosis rather than apoptosis, underlining the potential implications of relatively simple structural changes to phytochemical scaffolds [60]. Curcumin's clinical potential has been hampered by observations of poor bioavailability* in vivo*, sparking interest in chemical modifications to the scaffold that may improve such properties. One such derivative, EF24 (diphenyl difluoroketone), potently inhibits tumorigenesis in a mouse model of prostate cancer by downregulating NF-*κ*B and miRNA-21 expression [61]. High-throughput screening approaches have identified another curcumin analog, B82 ((1E,4E)-1,5-bis(5-bromo-2-ethoxyphenyl)penta-1,4-dien-3-one), which exhibits strong antitumor activity against non-small-cell lung cancer cells* in vivo* by inducing ER stress [62]. Similar approaches using resveratrol derivatives have shown that a number of parameters including VEGF inhibition, cytotoxicity, and inhibition of angiogenesis can be improved with side chain modifications to the parent structure [63]. Further research into the relationships between the structure and function of key molecular scaffolds and active side chains is ongoing and will inevitably lead to the discovery of novel drugs with enhanced target specificity.

## 7. Future Directions

Progress toward better therapeutic strategies for the treatment of gynecologic cancers will be reliant on steady innovation in the areas of prevention, detection, and treatment. The rapid advance of the computer age is providing more powerful software tools for bioinformatics approaches and meta-analyses. These are already yielding benefits, evident in the rapid emergence of publications in the field of systems biology in recent years. However, further integration is necessary for more comprehensive therapeutic solutions. For gynecologic cancers in particular, a severe lack of accurate biomarkers is hampering the effort to improve screening procedures. The identification of novel biomarkers requires a deeper understanding of the molecular mechanisms responsible and will drive the development of better diagnostic medical devices. Coupled with genotyping approaches, these advances will create the strong foundations necessary for a fully fledged era of personalized medicine.

Like all malignant neoplasms, gynecologic cancers arise not as a result of the disruption of a single cellular target, but only after a critical combination of mutations occurs that result in self-sufficient proliferation and survival signaling. The development of optimal treatment strategies will therefore need to shift away from the historical shotgun approaches of cytotoxic chemotherapy and focus on the identification of specific elements at play in each individual case. The beginning of the era of personalized medicine is being accelerated with the development of medical devices capable of genotyping patients quickly and affordably. However, these advances have yet to be matched with similar progress in the area of targeted cancer therapies for known mutations. A major factor for this discrepancy is undoubtedly the complexity of cell signaling pathways, emphasizing the need for continuing research into how these pathways culminate in the evasion of apoptotic signaling.

The exact details of how future personalized therapeutic approaches will operate remain to be determined by the wider medical community. It appears likely to involve individual genotyping for verified oncogenic mutations followed by targeted therapies tailored to an* in silico* evaluation of an optimal strategy. In order to curb the side effects of medication, it may also be advantageous to make the distinction between full, partial, and even subtle inhibition of certain molecular signaling components. In such cases, then, a larger molecular toolbox will provide a more versatile arsenal with which to untangle the molecular mechanisms responsible for each malignancy and eliminate the threat of proliferation. Phytochemicals represent a large and relatively undiscovered resource that can be exploited to supplement such a toolbox. The vast combinations of molecular structures that exist in the evolutionary inventory in some cases have been further enhanced by the fact that some structures have evolved specifically to disrupt molecular signaling pathways in animal cells for defense reasons. A number of lines of evidence support the hypothesis that dietary intake of specific phytochemicals imparts cancer chemopreventive effects, and this may be one factor in explaining regional variations in cancer incidence across the globe.

With further progress in the area of phytochemical-based approaches to gynecologic cancer therapy, it may soon become convention to treat such patients with capsules or infusions containing a cocktail of phytochemicals and rationally designed therapeutics tailored to the specifics of every individual patient. This will require further investments in high-throughput screening and other platform technologies to accelerate the hit-to-lead process and subsequent clinical trials. There are also tantalizing hints that the US FDA may be moving toward a system of regulatory approval for novel cancer therapeutics based solely on their molecular targets rather than the traditional indication-based approach. Such advances will no doubt enhance the versatility afforded to the pharmaceutical sector during strategic drug development decisions. This will contribute to improved overall patient survival and better quality of life through significant improvements in efficacy and the minimization of harmful side effects.
